# An expanded framework for biomolecular visualization in the classroom: Learning goals and competencies

**DOI:** 10.1002/bmb.20991

**Published:** 2016-08-03

**Authors:** Daniel R. Dries, Diane M. Dean, Laura L. Listenberger, Walter R.P. Novak, Margaret A. Franzen, Paul A. Craig

**Affiliations:** ^1^ Department of ChemistryJuniata CollegeHuntingdonPennsylvania; ^2^ Department of ChemistryUniversity of Saint JosephWest HartfordConnecticut; ^3^ Departments of Chemistry and BiologySt. Olaf CollegeNorthfieldMinnesota; ^4^ Department of ChemistryWabash CollegeCrawfordsvilleIndiana; ^5^ Center for BioMolecular ModelingMilwaukee School of EngineeringMilwaukeeWisconsin; ^6^ School of Chemistry and Materials ScienceRochester Institute of TechnologyRochesterNew York

**Keywords:** Assessment of educational activities, computers in research and teaching, curriculum design development and implementation, judging quality of macromolecular models, molecular graphics and representations, molecular visualization, teaching and learning techniques methods and approaches, skill development including cognitive skills, using modeling as a research tool for investigating teaching, visual literacy

## Abstract

A thorough understanding of the molecular biosciences requires the ability to visualize and manipulate molecules in order to interpret results or to generate hypotheses. While many instructors in biochemistry and molecular biology use visual representations, few indicate that they explicitly teach visual literacy. One reason is the need for a list of core content and competencies to guide a more deliberate instruction in visual literacy. We offer here the second stage in the development of one such resource for biomolecular three‐dimensional visual literacy. We present this work with the goal of building a community for online resource development and use. In the first stage, overarching themes were identified and submitted to the biosciences community for comment: atomic geometry; alternate renderings; construction/annotation; het group recognition; molecular dynamics; molecular interactions; monomer recognition; symmetry/asymmetry recognition; structure‐function relationships; structural model skepticism; and topology and connectivity. Herein, the overarching themes have been expanded to include a 12th theme (macromolecular assemblies), 27 learning goals, and more than 200 corresponding objectives, many of which cut across multiple overarching themes. The learning goals and objectives offered here provide educators with a framework on which to map the use of molecular visualization in their classrooms. In addition, the framework may also be used by biochemistry and molecular biology educators to identify gaps in coverage and drive the creation of new activities to improve visual literacy. This work represents the first attempt, to our knowledge, to catalog a comprehensive list of explicit learning goals and objectives in visual literacy. © 2016 by The International Union of Biochemistry and Molecular Biology, 45(1):69–75, 2017.

Biomolecular science courses are laden with images. Students encounter visual representations of cell membranes, organelles, macromolecules, and biochemical pathways. The way in which these structures are presented can vary greatly 1. For example, proteins may be represented as ball‐and‐stick models, space‐filling models, blobs or **“**Pac‐man**”** cartoons, molecular surfaces, or ribbons, with each representation intentionally selected for the way in which it presents particular structural information. Moreover, textbooks present these structures and pathways as two‐dimensional objects, often ignoring how to relate these two‐dimensional images to the three dimensions of the real world 2. Yet some biochemical concepts (*e.g*. structure–function relationships) require knowledge of three‐dimensional structure and, therefore, necessitate a way to visualize and extract meaning from structure 3.

Despite the large role of visualization in biochemistry and molecular biology (BMB) education, do we explicitly teach visual literacy or do we simply show images and expect students to infer biochemical principles? What impedes our explicit teaching of visual literacy? How do we distinguish between true visual literacy and a student's ability to simply mimic the language of the field through memorization of terminology? While many have raised these questions before, there remains no formal rubric by which BMB educators may assess their students' acquisition of these skills.

Here, we address this problem by creating an extensive list of learning goals and objectives for teaching visual literacy. We submit these resources to the greater BMB education community for discussion and critique. This framework provides instructors with a blueprint for creating or revising curricula that are in line with the recommendations that have been made at the national level 4, 5, 6. Furthermore, instructors may choose to share this list of learning objectives with their class so that students can chart their own progress in molecular visualization.

## Success in the Molecular Biosciences: A Problem of Cognitive Load?

Visuospatial reasoning skills are critical for student retention and success in the molecular biosciences 7. This stems from the fact that interpretation of the myriad visual representations of biomolecules is key to understanding biochemical function 8. Moreover, we instructors not only show static images to our students but we also expect students to manipulate these structures to make hypotheses and inferences about how the molecules function. For these and other reasons, visual literacy was deemed a “threshold concept” at an August 2013 ASBMB symposium on student‐centered education. However, the working group felt that due to its ubiquity, visual literacy skills should be woven throughout the BMB curriculum. Regardless, the group highlighted the need for explicit instruction in these critical skills for a complete education in the molecular life sciences 9. The work presented here addresses this need by explicitly identifying a set of learning goals and objectives against which an educator may map his/her instruction in molecular visualization.

Too often, we make the assumption that our students' mental images and their comprehension of these visualizations are similar to our own. However, recent research suggests that many students have “substantial limitations in their ability to recognize and interpret basic features of molecules” 10. Indeed, studies of the perceptions of novices *versus* experts have confirmed this disconnect, as novices grapple with both the concept and the mental image simultaneously 11. Cognitive load theory suggests that we can only retain seven elements simultaneously in our working memory 12. Experts are able to chunk information, such that “protein structure” (a single element) encompasses the entirety of amino acid sequence, N‐ and C‐termini, α‐helices, 3_10_ helices, parallel and antiparallel beta sheets, loops, disulfide bonds, hydrophobic core, and much more. For the novice, however, each of these concepts is a unique element and, therefore, together, these concepts overload working memory. Furthermore, novices are frequently mastering both vocabulary and concepts simultaneously and have limited prior knowledge on which to build, all of which lead to further cognitive overload 13. In order to allow our students to transition from novices to experts, we must therefore provide explicit and intentional training in molecular visualization 11, 14. Moreover, unless visual literacy is explicitly assessed, there exists the possibility that exam questions test “some unknown combination of students' conceptual biochemical understanding and their visual literacy” 15.

## Molecular Visualization in the Classroom: The Need for a Road Map

As technology has developed, a wide range of ways of viewing macromolecules has emerged, with many of us enthusiastically adopting these tools in our teaching (for a review, see ref. 16). Moreover, a recent survey found that visual literacy is taught in biochemistry courses using approaches that range from small group exercises and student projects to incorporation into lectures 16. Yet the most common use of visualization in the classroom is merely exposure (*i.e*. not the explicit instruction in, *e.g*. selecting a type of representation, manipulating a structure, or thinking about how that structure may be used in a biological context) 15.

Herráez and Costa state that biochemical visual literacy requires, among other things, “adequate assessment tests and programs” 17. Yet while educators clearly recognize the importance of molecular visualization and literacy, assessment of these skills is often lacking 14. This may be because it is difficult for instructors to assess the effectiveness of their teaching this topic 16, 18 or because instructors assume students are visually literate from previous coursework 15. Craig *et al*. [16] also reported that educators “would like to use more formal rubrics, but their development can be challenging since the outcomes of molecular visualization are diverse and difficult to interpret on a quantitative scale.” Furthermore, this same study cited the desire for a “central repository for molecular visualization activities, effective methods of assessment, rubrics…” as a common request from educators. This manuscript submits to the BMB community a list of learning goals and objectives as a step toward development of this repository.

Having identified the explicit need for such a resource, Bateman and Craig proposed an assessment rubric for biomacromolecular three‐dimensional literacy that included 11 overarching themes. In expanding on the work of Bateman and Craig, we applied the practice of backward design, grounding our work in what we expect students to know or do and subsequently identifying appropriate competencies 19, 20. The benefits of this approach are numerous 21, 22. Identification of learning objectives influences selection of appropriate assignments and assessment activities. Higher level assessment of student achievement can be implemented by clearly articulated learning objectives. Moreover, students are better able to recognize the importance of a particular assignment and make connections to course or discipline content when learning objectives are shared. “Unpacking” of this framework centered on several questions: What skills must be developed for biomacromolecular three‐dimensional literacy? What are the prerequisite skills that must be developed as stepping stones to proficiency, and at what point in a student's professional career should these skills be mastered? What tools and activities will allow students to gain proficiency? What evidence will demonstrate that students have mastered these milestones?

**Figure 1:**
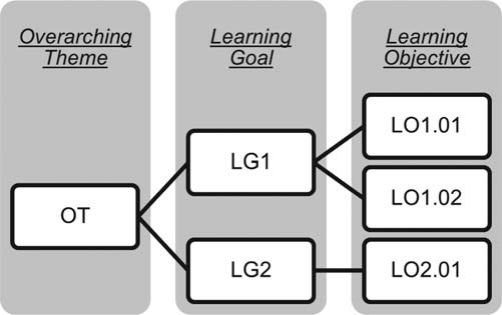
Organizational structure of the framework. An overarching theme (OT) is subdivided into learning goals (LG1, LG2, …), which in turn are further delineated into learning objectives (LO1.01, LO1.02, …).

**Figure 2:**
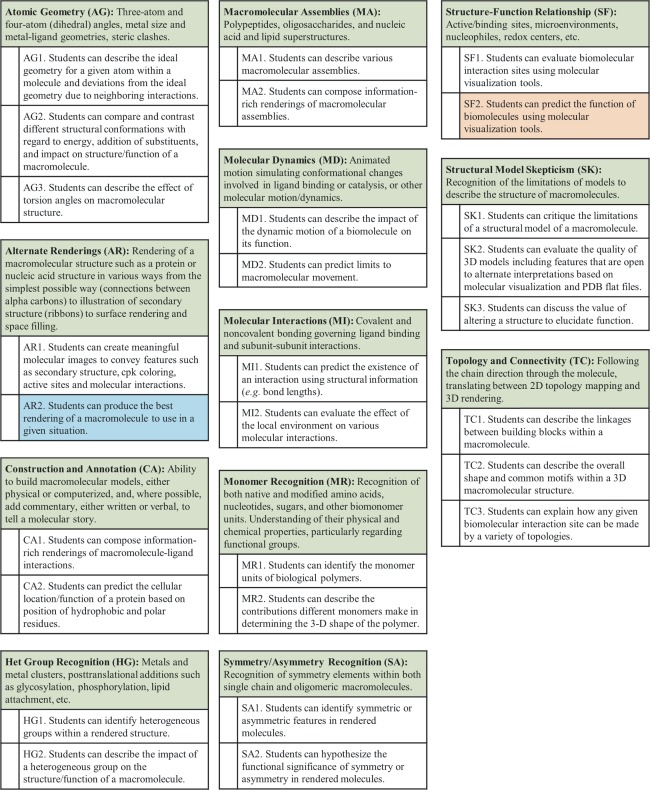
Levels 1 and 2 of the biomolecular visualization framework. Each block represents a different overarching theme. Below each theme is a list of learning goals. Highlighted are learning goals AR2 (expanded in Fig. 3) and SF2 (expanded in Fig. 4), which are discussed in the text to illustrate the utility of the framework. [Color figure can be viewed in the online issue, which is available at wileyonlinelibrary.com.]

**Figure 3:**
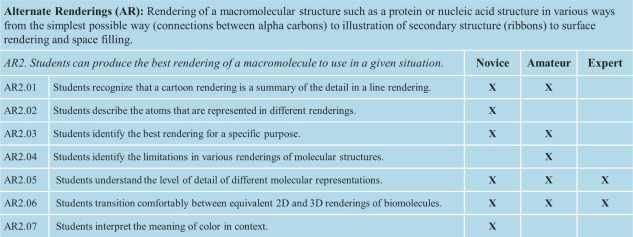
Expansion of learning goal AR2 from Fig. 2. Shown are the various learning objectives within the AR2 learning goal, along with the level of expertise at which they each would be addressed. [Color figure can be viewed in the online issue, which is available at wileyonlinelibrary.com.]

In this article and the accompanying web resource (http://cbm.msoe.edu/crest/molviz), we expand the 11 overarching themes first presented by Bateman and Craig and promulgate a set of 27 learning goals and more than 200 objectives for instruction of molecular visualization at the novice, amateur, and expert level (Fig. 1; see Table 1 for a description of terms used). For the sake of brevity, we sought to restrict our list to include only those learning goals and objectives that pertain to molecular visualization specifically, omitting those that do not require the three‐dimensional visualization of a macromolecule (*e.g*. ranking noncovalent interactions by energy). Each overarching theme (*e.g*. alternate renderings, AR) is subdivided into learning goals (Fig. 2). Each learning goal (*e.g*. AR2, Fig. 2, *blue*) is further delineated into learning objectives that are appropriate for the different levels of students: novice, amateur, and expert (Figs. 1 and 3). For example, learning objective AR2.05 has specific competencies at each level of student expertise (Fig. 3). In this way, BMB educators can identify specific themes, learning goals and objectives for the explicit instruction of biomolecular visual literacy. As an example, in the expansion of this framework we ourselves identified the need for a twelfth overarching theme—Macromolecular Assemblies—in order to capture those learning goals and objectives associated with visualization of supramolecular structures, such as the ribosome. Once articulated, the overarching themes, learning goals and objectives presented in our web resource may be used to guide the creation of appropriate assessments to test for acquisition of these visual literacy skills. As an example of the utility of this tool, we now walk through one learning goal that underlies the overarching theme of “Structure‐Function Relationship (SF).”

**Table 1:** Framework terminology and accompanying descriptions

**Molecular Visualization** in the context of this work is limited to macromolecular structures, although competencies at the novice level include recognition of monomers and oligomers.
**Learning Goals** are broad, long range outcomes of a course or program of study. They are general in nature. “Molecular visualization skills” is an example of a learning goal 23.
**Learning Objectives** “flesh out” the details of learning goals. They are brief, clear statements describing what students should know or do. Specific in nature, they describe the intended results of instruction. “Students should recognize alternate renderings of chemical structures” is an example of a learning objective 24.
**Competencies** identify the expected skill or knowledge level at the end of a course of instruction. Although the same learning objective may be appropriate for freshman, senior or graduate level courses, students are expected to demonstrate greater depth and breadth of knowledge as they progress. These differences in mastery are identified in competencies.
**Novices** are new to a field of study. For our purposes, we identify novices as college freshmen in an introductory biology course, although this may also include high school students in college‐level preparatory courses.
**Amateurs** have some experience in the field of study. Examples of amateurs would be students in cell biology or biochemistry or graduating seniors in biochemistry or molecular biology.
**Experts** are actively engaged in the field of study, including graduate students who have completed their coursework, post‐docs and researchers.

## Utility of the Biomolecular Visualization Framework

Visualization of macromolecules can elucidate the relationship of structure to function, a core concept in the molecular biosciences identified in Vision and Change 5. This big idea—that students should recognize that biological macromolecules derive function from structure—is one that is also explicitly identified in our expanded framework. The SF overarching theme was divided into two learning goals. Learning goal SF2 (Fig. 2, *salmon*) is unpacked below into a series of explicit learning objectives. This example illustrates the utility of the framework by showing how a single learning goal can be used with different competency expectations for various levels of expertise. In addition to describing the details of five SF learning objectives, an intersection between the larger overarching themes of SF and Molecular Dynamics is described. As the structure–function relationship is foundational to all of the areas of the framework, many of the learning goals and objectives are also relevant to the overarching theme of SF.

**Figure 4:**
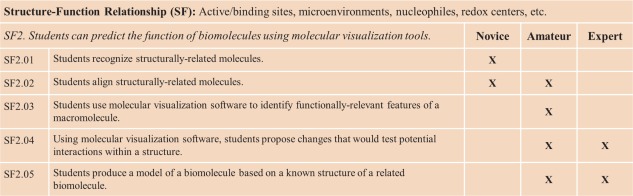
Learning objectives within learning goal SF2 from the SF overarching theme of Fig. 2. [Color figure can be viewed in the online issue, which is available at wileyonlinelibrary.com.]

Structure‐Function Relationship learning goal 2 (SF2) states that students can predict the function of biomolecules using molecular visualization tools. Toward this goal, SF2 specifies competencies at the levels of novice, amateur, and expert (Fig. 4). For example, the novice is able to recognize structurally related molecules (learning objective SF2.01) and do simple three‐dimensional alignments of related structures (SF2.02). The amateur can align multiple structures, including those that are more divergent (*e.g*. insertions, deletions). The amateur can also identify functionally relevant features of a given macromolecule (SF2.03); such inferences may be based on identifying interactions with a ligand(s) and/or surface properties of the macromolecule (*e.g*. polarity, contour, or electrostatic potential). Building on this competency, the amateur is able to propose alterations to change the function of the macromolecule (SF2.04). Such changes involve the alteration of functional groups with respect to size, shape, and polarity. The expert takes such predictions one step further, considering van der Waals packing, strength of dipoles and charges, and changes in the electrostatic potential. The expert also considers more subtle interactions, such as π‐stacking and induced dipoles. In the last learning objective, amateurs are able to do simple homology modeling with limited refinement, whereas experts are able to perform more sophisticated refinement of a resulting homology model (*e.g*. building loops or constructing chimaeras from two or more models) (SF2.05). In this way, a single learning goal used with differing competencies allows an instructor to tailor the course material to the targeted level of expertise with scaled learning objectives.

Finally, intersections between the different overarching themes can assist an instructor in creating meaningful connections for students. For example, a learning goal in the SF overarching theme may intersect with one from the Molecular Dynamics theme to tie movement to function. The Molecular Dynamics overarching theme relates to animated motion simulating conformational changes involved in ligand binding or catalysis or other molecular motions/dynamics. The MD1 learning goal states that students can describe the impact of the dynamic motion of a biomolecule on its function. The ability of a student to recognize the importance of movement to function hinges on that student's understanding of the structure–function relationship. Thus, instructors can first establish mastery of the structure–function relationship and then expand that mastery to include movement. Students will build a solid understanding by connecting the necessity of the movement to the molecule's function to their previous knowledge about how the function is derived from the molecule's structure. The structure–function relationship is a unifying theme in the study of macromolecular visualization. Hence the SF learning goal has connections to many of the other learning goals described in the framework. This interconnectivity of visualization learning goals allows instructors to interleave concepts and layer complexity over time, both important strategies for mastery 23.

## Using the Framework to Develop Assessments

One use of the framework is to assist faculty in the development of molecular visualization assignments that capture both the breadth and depth of needed skills. Here, we provide an example of how the learning objectives under the topic of AR can lead to questions for student practice or assessment. We focus on Alternate Rendering learning goal AR1: “Students can create meaningful molecular images to convey features such as secondary structure, CPK coloring, active sites and molecular interactions.” As educators we might hope that our students are developing this skill, but how can we be sure? On the website we propose ten learning objectives for this goal for which an educator can consider creating specific assessments. Learning objective AR1.04 states, “students can infer information from rendering a structure in different ways.” A sample assessment at the amateur level could be the following:

Given the structure of 5‐methylthioribose 1‐phosphate (MTRu‐1‐P) isomerase bound to MTRu‐1‐P (PDB ID: 2yvk chain A), use Jmol (or another molecular visualization tool such as Chimera or PyMOL) to identify the amino acids in the protein that interact with the ligand.

**Figure 5:**
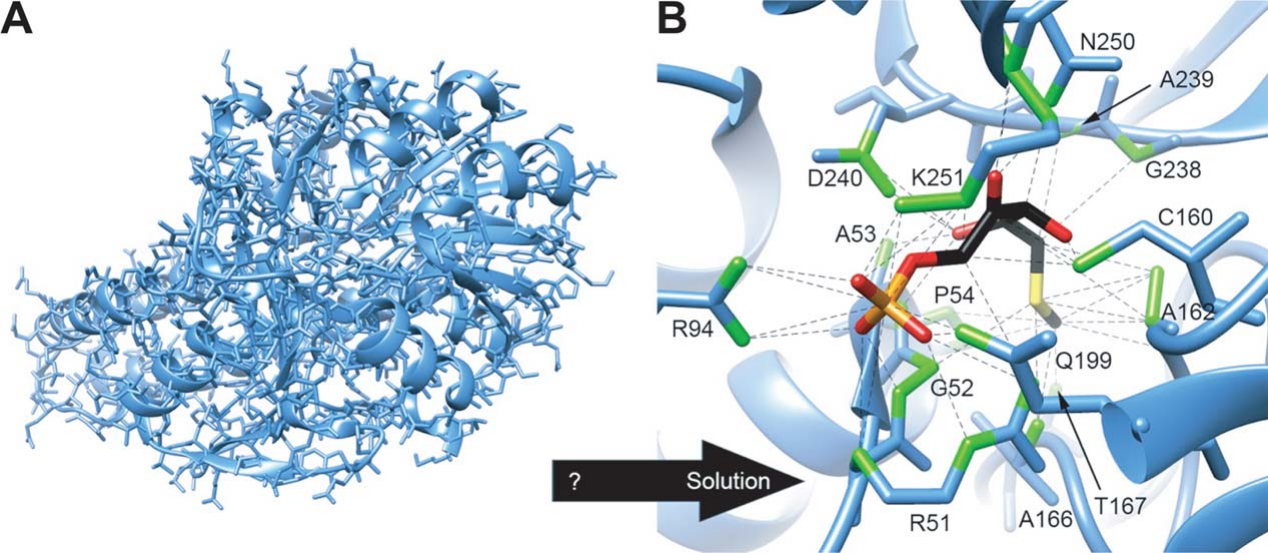
(A) Overview of 5‐methylthioribose 1‐phosphate isomerase (MTRu‐1‐P isomerase, light blue). Ribbon diagram with all side chains shown in stick representation, showing the complexity of the full molecule. (B) How a student might find the answer to the problem. Ribbon diagram of 5‐methylthioribose 1‐phosphate (MTRu‐1‐P) isomerase with side chains contacting the MTRu‐1‐P ligand shown in stick representation. The ligand is shown in CPK coloring. Atoms contacting the ligand are shown in green, and contacts are indicated with dashed lines. (Image made with UCSF Chimera.)

Students would then be expected to use the software to identify the residues that come into contact with the ligand, including residues involved in van der Waals and hydrogen bonding. In this case the answer includes a number of residues differing in size, charge, and polarity (Fig. 5). This same problem could also be used to assess Structure Function learning goal SF1.01, “Students can distinguish protein, cofactors and small molecule ligands or substrates” as students would need to identify the ligand in order to successfully complete the assessment. In this way, the learning goals and objectives for a given level of student can be used to identify an appropriate assessment tool to demonstrate visual literacy.

## An Invitation

The goal of introducing students to visualization of macromolecules is to improve the understanding of the functions of these critical biological components. The framework illustrates the learning goals and objectives necessary to achieve improved student comprehension of molecular visualization. Each of the learning goals is divided into teachable objectives scaled to varying student skill levels. The instructor can use the framework to improve student learning by ensuring the coverage of multiple aspects of visualization, as well as to illustrate the connections between differing overarching themes. The revised framework will facilitate the explicit instruction of visual literacy in the classroom by providing specific goals to improve the mastery of visualization of macromolecules and thus improve the student's ability to describe the function of differing classes of macromolecules. This framework is the product of a collaboration from a diverse group of educators. As the next step toward a broader effort, we invite the BMB community to use, evaluate, critique, and indeed help improve upon the work presented here. In this way, we wish to grow a community of practice that is devoted to facilitating the constructive use of biomolecular visualization in the classroom. Please visit http://cbm.msoe.edu/crest/molviz to see the entire framework and for the opportunity to join our working group on constructing a guide for visualization literacy. Together, we will identify appropriate competencies at each level to inform the creation of student‐centered instructional materials and assessments that will enable students to develop molecular visualization literacy and, therefore, demonstrate greater competence in the molecular life sciences as a whole.
